# Supplementary material for: Effectiveness of Cell-Free and Concentrated Ascites Reinfusion Therapy in the Treatment of Malignancy-Related Ascites: A Systematic Review and Meta-Analysis
Source: Cancers (Basel). 2021 Sep 29;13(19):4873. doi: 10.3390/cancers13194873 (PMC8508032; doi:10.3390/cancers13194873)
Supplement: Supplementary file 1 [file cancers-13-04873-s001.zip › cancers-1343297-supplementary.pdf]

cancers MDPI

Table S1. Outcomes of each study enrolled.

Authors and Year of Publication		Outcomes
Hanada	2018	Primary outcomes: time to next paracentesis and patient-reported symptoms. Secondary outcomes: adverse events, laboratory findings, and changes in physical findings.
Hanafusa	2017	Patients' characteristics; CART conditions; laboratory findings; adverse events et al.
Ito	2015	The severity of patients’ malignancy-related symptoms et al.
Ito	2020	Ascitic cytokines; body temperature and laboratory findings et al.
Iwaki	2018	Laboratory findings, symptoms, and adverse events et al.
Kawata	2019	Patients' characteristics and physiological changes; the number of ascites tumor cell
Maeda	2014	Laboratory findings, patients' characteristics et al.
Matsusaki	2020	Procedure information of CART, change of vital signs, alleviating signs/symptoms et al.
Nagata	2020	Changes of PS, Laboratory findings, adverse events; the response of chemotherapy et al.
Ohta	2017	Changes of PS, Laboratory findings, symptoms et al.
Togami	2014	Changes of PS, Laboratory findings, adverse events et al.
Ueda	2012	Laboratory findings, patients' characteristics; adverse events
Wang	2015	Laboratory findings, Adverse events et al.
Yamaguchi	2015	OS of chemotherapy patients with or without CART et al.
Yamamoto	2021	CART condition, Efficacy, and safety aspects of CART

PS: performance status; CART: cell-free and concentrated ascites reinfusion therapy; OS overall survival.

Table S2. Evaluation of the studies by modified-Newcastle-Ottawa Scale.

Authors and Year of Publication		Selection			Outcome			Total Score
		a	b	c	d	e	f	
Hanada	2018	*	*	*	*	*	*	******
Hanafusa	2017	-	*	*	*	*	*	*****
Ito	2015	-	*	*	*	*	*	*****
Ito	2020	*	*	*	*	*	-	*****
Iwaki	2018	*	*	*	-	*	-	****
Kawata	2019	*	*	*	*	*	*	******
Maeda	2014	*	*	*	*	*	*	******
Matsusaki	2020	*	*	*	*	*	*	******
Nagata	2020	*	*	*	*	*	*	******
Ohta	2017	*	*	*	*	*	-	*****
Togami	2014	*	*	*	*	*	*	******
Ueda	2012	*	*	*	*	*	*	******
Wang	2015	*	*	*	*	*	*	******
Yamaguchi	2015	*	*	*	*	*	*	******
Yamamoto	2021	*	*	*	*	*	*	******

(a) Representativeness of the exposed cohort; (b) Ascertainment of exposure; (c) Demonstration that outcome of interest was not present at start of study; (d) Assessment of outcome; (e) Was follow-up long enough for outcomes to occur; (f) Adequacy of follow up of cohorts.

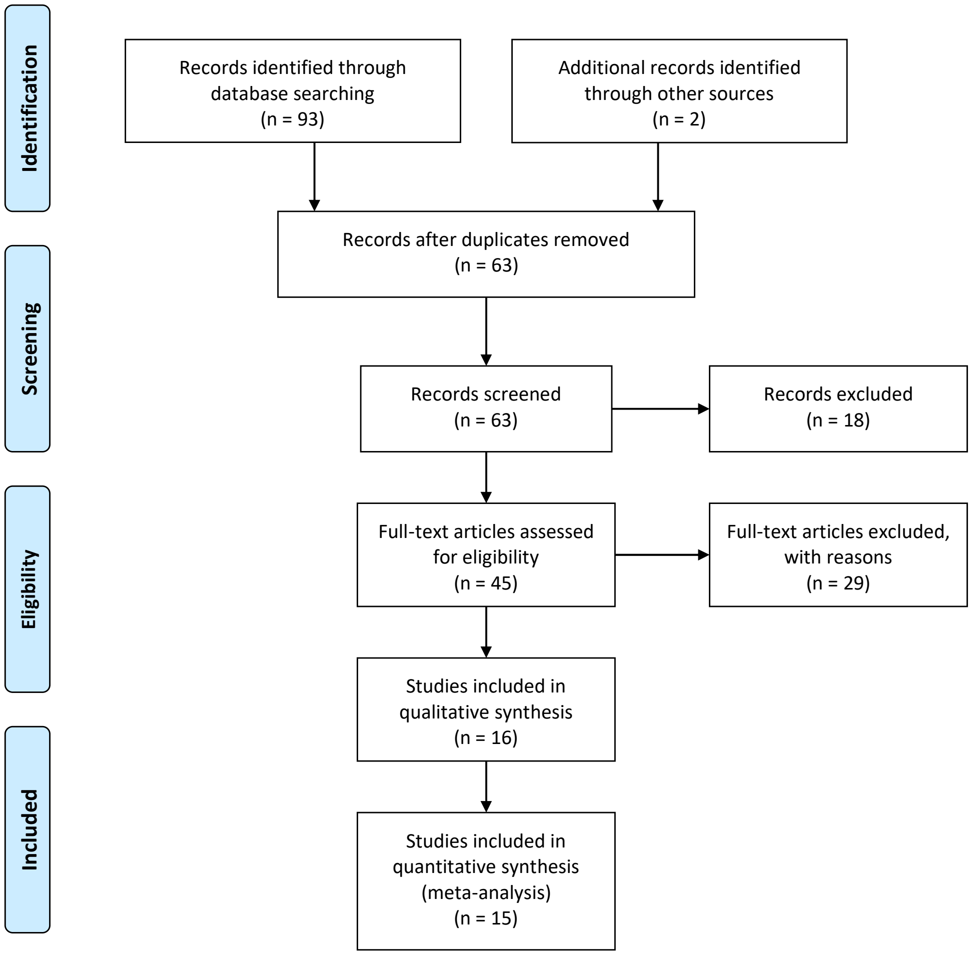

Figure S1. PRISMA flow chart for study selection.

Study	Mean Difference	SE	Weight	Mean Difference IV, Random, 95% CI
Hanada 2018	5.86	0.18	7.2%	5.86 [5.51, 6.21]
Hanafusa 2017	3.71	0.09	7.3%	3.71 [3.53, 3.89]
Ito 2014	3.2	0.14	7.2%	3.20 [2.93, 3.47]
Ito 2020	3.21	0.13	7.2%	3.21 [2.96, 3.46]
Iwaki 2018	7	0.42	6.8%	7.00 [6.18, 7.82]
Kawata 2019	2.94	0.12	7.2%	2.94 [2.70, 3.18]
Maeda 2014	4.01	0.18	7.2%	4.01 [3.66, 4.36]
Matsusaki 2020	6.2	0.06	7.3%	6.20 [6.08, 6.32]
Nagata 2020	4	0.2	7.2%	4.00 [3.61, 4.39]
Togami 2014	3.19	0.28	7.1%	3.19 [2.64, 3.74]
Ueda 2012	3.29	0.16	7.2%	3.29 [2.98, 3.60]
Wang 2015	7.73	0.45	6.7%	7.73 [6.85, 8.61]
Yamaguchi 2015	3.06	0.11	7.3%	3.06 [2.84, 3.28]
Yamamoto 2021	3.01	0.18	7.2%	3.01 [2.66, 3.36]
**Total (95% CI)**			**100.0%**	**4.29 [3.47, 5.11]**

Heterogeneity: $Tau^2 = 2.40$; $Chi^2 = 1705.81$, df = 13 ($P < 0.00001$); $I^2 = 99\%$
Test for overall effect: Z = 10.26 ($P < 0.00001$)

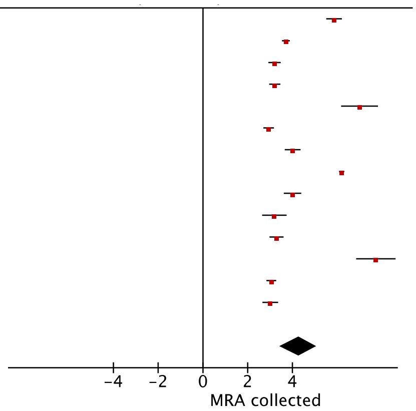

Figure S2. The mean volume of MRA collected.

Study	Mean Difference	SE	Weight	Mean Difference IV, Random, 95% CI
Hanada 2018	0.764	0.031	9.1%	0.76 [0.70, 0.82]
Hanafusa 2017	0.491	0.017	9.3%	0.49 [0.46, 0.52]
Ito 2014	0.302	0.015	9.3%	0.30 [0.27, 0.33]
Ito 2020	0.299	0.014	9.3%	0.30 [0.27, 0.33]
Kawata 2019	0.272	0.012	9.3%	0.27 [0.25, 0.30]
Maeda 2014	0.561	0.029	9.1%	0.56 [0.50, 0.62]
Matsusaki 2020	0.61	0.006	9.4%	0.61 [0.60, 0.62]
Ohta 2017	0.538	0.064	8.3%	0.54 [0.41, 0.66]
Togami 2014	0.92	0.062	8.4%	0.92 [0.80, 1.04]
Wang 2015	0.334	0.014	9.3%	0.33 [0.31, 0.36]
Yamaguchi 2015	0.392	0.027	9.2%	0.39 [0.34, 0.44]
**Total (95% CI)**			**100.0%**	**0.49 [0.39, 0.60]**

Heterogeneity: $Tau^2 = 0.03$; $Chi^2 = 1340.20$, df = 10 (P < 0.00001); $I^2 = 99\%$
Test for overall effect: Z = 8.95 (P < 0.00001)

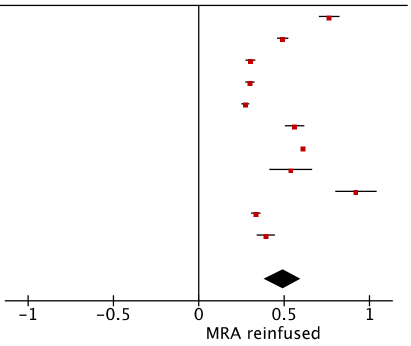

Figure S3. Mean volume of MRA reinfused.

Study	Mean Difference	SE	Weight	Mean Difference IV, Random, 95% CI
Hanafusa 2017	66.8	1.73	14.3%	66.80 [63.41, 70.19]
Ito 2014	93.1	5.16	12.3%	93.10 [82.99, 103.21]
Ito 2020	91.3	4.78	12.6%	91.30 [81.93, 100.67]
Kawata 2019	85	4.84	12.6%	85.00 [75.51, 94.49]
Maeda 2014	75	4.17	13.0%	75.00 [66.83, 83.17]
Matsusaki 2020	67.3	0.94	14.5%	67.30 [65.46, 69.14]
Wang 2015	161.2	11.7	7.5%	161.20 [138.27, 184.13]
Yamaguchi 2015	85.5	4.16	13.1%	85.50 [77.35, 93.65]
**Total (95% CI)**			**100.0%**	**86.12 [77.08, 95.17]**

Heterogeneity: $Tau^2 = 145.94$; $Chi^2 = 137.24$, df = 7 (P < 0.00001); $I^2 = 95\%$
Test for overall effect: Z = 18.66 (P < 0.00001)

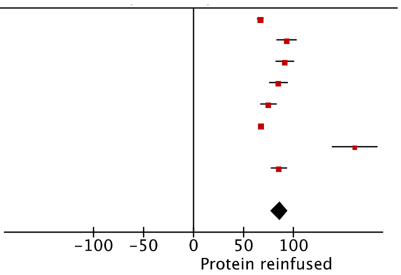

Figure S4. Mean amount of total protein reinfused.

Study	After CART Mean	SD	Total	Before CART Mean	SD	Total	Weight	Mean Difference IV, Random, 95% CI
Hanada 2018	3.32	0.7	104	3.1	0.5	104	9.1%	0.22 [0.05, 0.39]
Hanafusa 2017	2.9	0.9	350	2.5	0.5	350	9.8%	0.40 [0.29, 0.51]
Iwaki 2018	2.6	0.5	39	2.5	0.5	39	8.4%	0.10 [−0.12, 0.32]
Kawata 2019	2.6	0.5	47	2.4	0.4	47	8.9%	0.20 [0.02, 0.38]
Maeda 2014	2.8	0.8	51	2.8	0.8	51	7.1%	0.00 [−0.31, 0.31]
Matsusaki 2020	2.52	0.55	2224	2.65	0.59	2224	10.3%	−0.13 [−0.16, −0.10]
Nagata 2020	2.79	0.62	100	2.65	0.54	100	9.2%	0.14 [−0.02, 0.30]
Ohta 2017	2.9	0.5	12	2.8	0.4	12	6.4%	0.10 [−0.26, 0.46]
Togami 2014	3	0.5	12	2.8	0.6	12	5.4%	0.20 [−0.24, 0.64]
Ueda 2012	2.39	0.73	57	2.34	0.64	57	7.9%	0.05 [−0.20, 0.30]
Wang 2015	2.66	0.73	58	2.4	0.56	58	8.2%	0.26 [0.02, 0.50]
Yamaguchi 2015	2.6	0.6	127	2.5	0.5	127	9.5%	0.10 [−0.04, 0.24]
**Total (95% CI)**			**3181**			**3181**	**100.0%**	**0.14 [−0.01, 0.28]**

Heterogeneity: $Tau^2 = 0.06$; $Chi^2 = 126.54$, df = 11 (P < 0.00001); $I^2 = 91\%$
Test for overall effect: Z = 1.81 (P = 0.07)

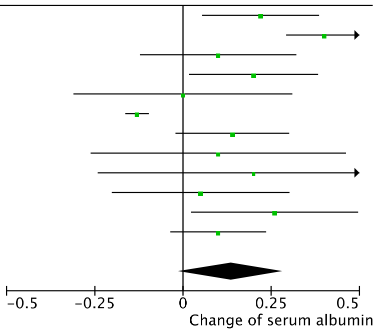

Figure S5. Mean amount of albumin reinfused.

Study	Mean Difference	SE	Weight	Mean Difference IV, Random, 95% CI
Hanada 2018 †	27	1.37	25.3%	27.00 [24.31, 29.69]
Hanafusa 2017 ‡	16.5	1.78	24.2%	16.50 [13.01, 19.99]
Maeda 2014 ‡	16.6	1.36	25.4%	16.60 [13.93, 19.27]
Ueda 2012 †	22.5	1.44	25.1%	22.50 [19.68, 25.32]
**Total (95% CI)**			**100.0%**	**20.69 [15.62, 25.77]**

Heterogeneity: $Tau^2 = 24.62$; $Chi^2 = 37.12$, df = 3 (P < 0.00001); $I^2 = 92\%$
Test for overall effect: Z = 7.99 (P < 0.00001)

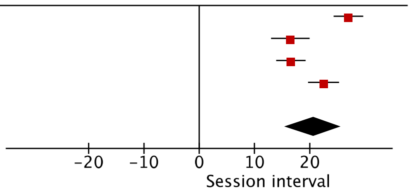

†: data was calculated from figure, paracentesis interval; ‡: session interval

Figure S6. Median time to next paracentesis.

Study	After CART Mean	SD	Total	Before CART Mean	SD	Total	Weight	Mean Difference IV, Random, 95% CI
Hanada 2018	6.3	1.1	104	6.1	0.8	104	12.4%	0.20 [−0.06, 0.46]
Hanafusa 2017	6.6	1.2	350	5.9	0.9	350	12.8%	0.70 [0.54, 0.86]
Iwaki 2018	5.7	0.7	39	5.6	0.7	39	12.1%	0.10 [−0.21, 0.41]
Kawata 2019	5.59	0.7	47	5.32	0.79	47	12.2%	0.27 [−0.03, 0.57]
Maeda 2014	5.65	0.61	51	5.5	0.57	51	12.5%	0.15 [−0.08, 0.38]
Matsusaki 2020	5.86	0.79	2224	6.33	0.79	2224	13.0%	−0.47 [−0.52, −0.42]
Wang 2015	5.39	0.76	58	5.19	0.63	58	12.4%	0.20 [−0.05, 0.45]
Yamaguchi 2015	5.6	0.9	127	5.3	0.8	127	12.6%	0.30 [0.09, 0.51]
**Total (95% CI)**			**3000**			**3000**	**100.0%**	**0.18 [−0.23, 0.59]**

Heterogeneity: $Tau^2 = 0.33$; $Chi^2 = 301.84$, df = 7 (P < 0.00001); $I^2 = 98\%$
Test for overall effect: Z = 0.86 (P = 0.39)

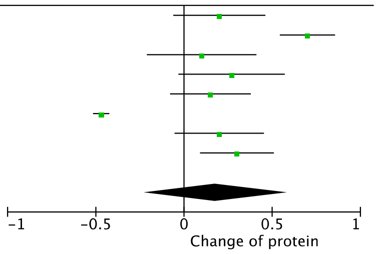

Figure S7. Change of total protein after CART.

Study	After CART Mean	SD	Total	Before CART Mean	SD	Total	Weight	Mean Difference IV, Random, 95% CI
Hanada 2018	49.8	15.5	104	53.1	12.1	104	15.3%	−3.30 [−7.08, 0.48]
Hanafusa 2017	52.4	14.6	350	56	15.1	350	45.1%	−3.60 [−5.80, −1.40]
Iwaki 2018	58.7	15.9	39	63.8	16.6	39	4.2%	−5.10 [−12.31, 2.11]
Kawata 2019	51.7	9.6	47	54.7	10.3	47	13.5%	−3.00 [−7.03, 1.03]
Ueda 2012	49.7	8.8	57	52.6	8.4	57	21.9%	−2.90 [−6.06, 0.26]
**Total (95% CI)**			**597**			**597**	**100.0%**	**−3.38 [−4.86, −1.90]**

Heterogeneity: $Tau^2 = 0.00$; $Chi^2 = 0.38$, df = 4 (P = 0.98); $I^2 = 0\%$
Test for overall effect: Z = 4.49 (P < 0.00001)

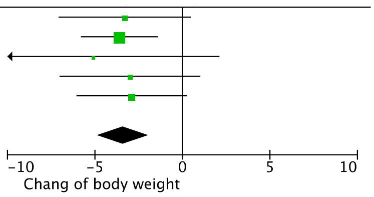

Figure S8. Change of creatine after CART.

Study	After CART Mean	SD	Total	Before CART Mean	SD	Total	Weight	Mean Difference IV, Random, 95% CI
Hanada 2018	75.8	35.6	104	66	29	104	3.2%	9.80 [0.98, 18.62]
Hanafusa 2017	71.3	30.5	350	66.6	27.9	350	13.4%	4.70 [0.37, 9.03]
Kawata 2019	79.49	28.45	47	72.04	28.87	47	1.9%	7.45 [−4.14, 19.04]
Matsusaki 2020	73.3	30.8	2224	66.1	29	2224	81.5%	7.20 [5.44, 8.96]
**Total (95% CI)**			**2725**			**2725**	**100.0%**	**6.95 [5.37, 8.54]**

Heterogeneity: $Tau^2 = 0.00$; $Chi^2 = 1.52$, df = 3 (P = 0.68); $I^2 = 0\%$
Test for overall effect: Z = 8.59 (P < 0.00001)

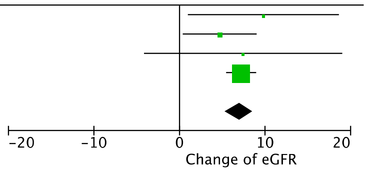

Figure S9. Change of eGFR after CART.

Study	Mean Difference	SE	Weight	Mean Difference IV, Random, 95% CI
Nagata 2020	0.05	0.022	35.3%	0.05 [0.01, 0.09]
Ohta 2017	0.27	0.045	31.9%	0.27 [0.18, 0.36]
Ueda 2012	0.2	0.04	32.8%	0.20 [0.12, 0.28]
**Total (95% CI)**			**100.0%**	**0.17 [0.03, 0.31]**

Heterogeneity: $Tau^2 = 0.01$; $Chi^2 = 25.10$, df = 2 (P < 0.00001); $I^2 = 92\%$
Test for overall effect: Z = 2.37 (P = 0.02)

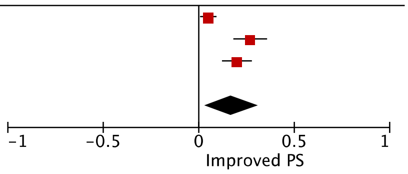

Figure S10. Improved performance status after CART.

Study	After CART Mean	SD	Total	Before CART Mean	SD	Total	Weight	Mean Difference IV, Random, 95% CI
Hanada 2018	36.7	0.3	104	36.6	0.4	104	21.5%	0.10 [0.00, 0.20]
Ito 2014	37.1	0.5	100	36.8	0.4	100	20.9%	0.30 [0.17, 0.43]
Kawata 2019	37.2	0.6	47	36.8	0.4	47	18.7%	0.40 [0.19, 0.61]
Yamaguchi 2015	37	0.5	127	36.7	0.4	127	21.2%	0.30 [0.19, 0.41]
Yamamoto 2021	37.6	0.8	49	36.6	0.3	49	17.7%	1.00 [0.76, 1.24]
**Total (95% CI)**			**427**			**427**	**100.0%**	**0.40 [0.18, 0.62]**

Heterogeneity: $Tau^2 = 0.06$; $Chi^2 = 49.78$, df = 4 (P < 0.00001); $I^2 = 92\%$
Test for overall effect: Z = 3.58 (P = 0.0003)

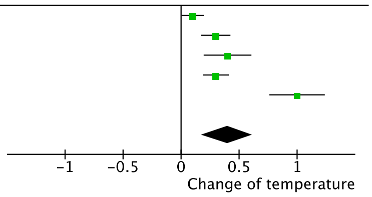

Figure S11. Change of body temperature after CART.

Study	After CART Mean	After CART SD	After CART Total	Before CART Mean	Before CART SD	Before CART Total	Weight	Mean Difference IV, Random, 95% CI
Hanada 2018	27.6	12.4	104	32	14.6	104	14.0%	−4.40 [−8.08, −0.72]
Hanafusa 2017	22.8	13.8	350	23.7	14.2	350	19.3%	−0.90 [−2.97, 1.17]
Ito 2014	28.14	14.58	81	30.79	16.83	81	10.8%	−2.65 [−7.50, 2.20]
Ito 2020	22.27	14.26	123	31.65	15.09	123	14.1%	−9.38 [−13.05, −5.71]
Iwaki 2018	27	12.8	39	32.3	13.6	39	8.7%	−5.30 [−11.16, 0.56]
Kawata 2019	36.9	17.9	47	42.4	20.9	47	5.8%	−5.50 [−13.37, 2.37]
Wang 2015	27.4	12.9	58	33.1	16.8	58	9.5%	−5.70 [−11.15, −0.25]
Yamaguchi 2015	32.7	10.2	127	36.5	10.1	127	17.9%	−3.80 [−6.30, −1.30]
**Total (95% CI)**			**929**			**929**	**100.0%**	**−4.39 [−6.57, −2.22]**

Heterogeneity: $Tau^2 = 5.27$; $Chi^2 = 17.51$, df = 7 (P = 0.01); $I^2 = 60\%$
Test for overall effect: Z = 3.96 (P < 0.0001)

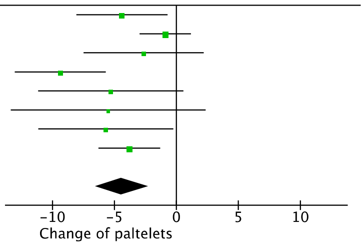

Figure S12. Change of platelets after CART.
